# Supplementary material for: The effect of captivity on the skin microbial symbionts in three Atelopus species from the lowlands of Colombia and Ecuador
Source: PeerJ. 2017 Jul 31;5:e3594. doi: 10.7717/peerj.3594 (PMC5541920; doi:10.7717/peerj.3594)
Supplement: Supplemental Information 1 — The table summarize the information of each isolate. [file peerj-05-3594-s001.pdf]

Sequence_ID	Strain_ID	Country	Host	Isolation_source	Organism	Submission_number	Genbank_number
ABZ.EC002	E1 - 2	Ecuador	*Atelopus elegans*	Wild Host	*Enterobacter sp.*	SUB2577474	KY938077
ABZ.EC003	E1 - 3	Ecuador	*Atelopus elegans*	Wild Host	*Klebsiella sp.*	SUB2577474	KY938078
ABZ.EC004	E1 - 4	Ecuador	*Atelopus elegans*	Wild Host	*Stenotrophomonas maltophilia*	SUB2577474	KY938079
ABZ.EC005	E1 - 5	Ecuador	*Atelopus elegans*	Wild Host	*Yokenella regensburgei*	SUB2577474	KY938080
ABZ.EC006	E1 - 6	Ecuador	*Atelopus elegans*	Wild Host	*Blastomonas natatoria*	SUB2577474	KY938081
ABZ.EC007	E1 - 7	Ecuador	*Atelopus elegans*	Wild Host	*Raoultella ornithinolytica*	SUB2577474	KY938082
ABZ.EC008	E1 - 8	Ecuador	*Atelopus elegans*	Wild Host	*Pseudomonas sp.*	SUB2577474	KY938083
ABZ.EC009	E1 - 9	Ecuador	*Atelopus elegans*	Wild Host	*Blastomonas natatoria*	SUB2577474	KY938084
ABZ.EC012	E2 - 1	Ecuador	*Atelopus elegans*	Wild Host	*Erwinia amylovora*	SUB2577474	KY938085
ABZ.EC013	E2 - 2	Ecuador	*Atelopus elegans*	Wild Host	*Erwinia amylovora*	SUB2577474	KY938086
ABZ.EC015	E2 - 4	Ecuador	*Atelopus elegans*	Wild Host	*Pseudomonas fulva*	SUB2577474	KY938087
ABZ.EC016	E2 - 5	Ecuador	*Atelopus elegans*	Wild Host	*Kluyvera ascorbata*	SUB2577474	KY938088
ABZ.EC017	E2 - 6	Ecuador	*Atelopus elegans*	Wild Host	*Staphylococcus capitis*	SUB2577474	KY938089
ABZ.EC019	E3 - 3	Ecuador	*Atelopus elegans*	Wild Host	*Pseudomonas sp.*	SUB2577474	KY938090
ABZ.EC020	E3 - 4	Ecuador	*Atelopus elegans*	Wild Host	*Citrobacter freundii*	SUB2577474	KY938091
ABZ.EC021	E3 - 5	Ecuador	*Atelopus elegans*	Wild Host	*Klebsiella oxytoca*	SUB2577474	KY938092
ABZ.EC022	E3 - 6	Ecuador	*Atelopus elegans*	Wild Host	*Novosphingobium subterraneum*	SUB2577474	KY938093
ABZ.EC023	E3 - 7	Ecuador	*Atelopus elegans*	Wild Host	*Caulobacter crescentus*	SUB2577474	KY938094
ABZ.EC024	E3 - 8	Ecuador	*Atelopus elegans*	Wild Host	*Brevundimonas aurantiaca*	SUB2577474	KY938095
ABZ.EC026	E4 - 2	Ecuador	*Atelopus elegans*	Wild Host	*Caulobacter vibrioides*	SUB2577474	KY938096
ABZ.EC027	E4 - 3	Ecuador	*Atelopus elegans*	Wild Host	*Duganella sp.*	SUB2577474	KY938097
ABZ.EC028	E4 - 4	Ecuador	*Atelopus elegans*	Wild Host	*Enterobacter sp.*	SUB2577474	KY938098
ABZ.EC029	E4 - 5	Ecuador	*Atelopus elegans*	Wild Host	*Microbacterium testaceum*	SUB2577474	KY938099
ABZ.EC030	E4 - 6	Ecuador	*Atelopus elegans*	Wild Host	*Luteibacter rhizovicinus*	SUB2577474	KY938100
ABZ.EC031	E4 - 7	Ecuador	*Atelopus elegans*	Wild Host	*Blastomonas natatoria*	SUB2577474	KY938101
ABZ.EC032	E4 - 8	Ecuador	*Atelopus elegans*	Wild Host	*Novosphingobium subterraneum*	SUB2577474	KY938102
ABZ.EC033	E4 - 9	Ecuador	*Atelopus elegans*	Wild Host	*Novosphingobium subterraneum*	SUB2577474	KY938103
ABZ.EC034	E5 - 1	Ecuador	*Atelopus elegans*	Wild Host	*Stenotrophomonas sp.*	SUB2577474	KY938104
ABZ.EC035	E5 - 2	Ecuador	*Atelopus elegans*	Wild Host	*Novosphingobium subterraneum*	SUB2577474	KY938105
ABZ.EC036	E5 - 3	Ecuador	*Atelopus elegans*	Wild Host	*Blastomonas natatoria*	SUB2577474	KY938106
ABZ.EC037	E5 - 4	Ecuador	*Atelopus elegans*	Wild Host	*Sphingomonas sp.*	SUB2577474	KY938107
ABZ.EC038	E5 - 5	Ecuador	*Atelopus elegans*	Wild Host	*Rhizobium sp.*	SUB2577474	KY938108
ABZ.EC039	E5 - 6	Ecuador	*Atelopus elegans*	Wild Host	*Pseudomonas putida*	SUB2577474	KY938109
ABZ.EC040	E5 - 7	Ecuador	*Atelopus elegans*	Wild Host	*Novosphingobium subterraneum*	SUB2577474	KY938110
ABZ.EC041	E6 - 1	Ecuador	*Atelopus elegans*	Captive Host	*Pseudomonas putida*	SUB2577474	KY938111
ABZ.EC042	E6 - 2	Ecuador	*Atelopus elegans*	Captive Host	*Enterobacter asburiae*	SUB2577474	KY938112
ABZ.EC044	E6 - 4	Ecuador	*Atelopus elegans*	Captive Host	*Rhizobium sp.*	SUB2577474	KY938113
ABZ.EC045	E6 - 5	Ecuador	*Atelopus elegans*	Captive Host	*Sphingomonas paucimobilis*	SUB2577474	KY938114
ABZ.EC046	E6 - 6	Ecuador	*Atelopus elegans*	Captive Host	*Pseudomonas fulva*	SUB2577474	KY938115
ABZ.EC047	E7 - 1	Ecuador	*Atelopus elegans*	Captive Host	*Massilia sp.*	SUB2577474	KY938116
ABZ.EC048	E7 - 2	Ecuador	*Atelopus elegans*	Captive Host	*Pseudomonas vranovensis*	SUB2577474	KY938117
ABZ.EC049	E7 - 3	Ecuador	*Atelopus elegans*	Captive Host	*Sphingobium yanoikuyae*	SUB2577474	KY938118
ABZ.EC050	E7 - 4	Ecuador	*Atelopus elegans*	Captive Host	*Blastomonas natatoria*	SUB2577474	KY938119

ABZ.EC051	E7 - 5	Ecuador	*Atelopus elegans*	Captive Host	*Pelomonas puraquae*	SUB2577474	KY938120
ABZ.EC052	E7 - 6	Ecuador	*Atelopus elegans*	Captive Host	*Pseudomonas sp.*	SUB2577474	KY938121
ABZ.EC053	E8 - 1	Ecuador	*Atelopus elegans*	Captive Host	*Pseudomonas vranovensis*	SUB2577474	KY938122
ABZ.EC054	E8 - 2	Ecuador	*Atelopus elegans*	Captive Host	*Pseudomonas sp.*	SUB2577474	KY938123
ABZ.EC055	E8 - 3	Ecuador	*Atelopus elegans*	Captive Host	*Pseudomonas sp.*	SUB2577474	KY938124
ABZ.EC056	E8 - 4	Ecuador	*Atelopus elegans*	Captive Host	*Pseudomonas saccharophila*	SUB2577474	KY938125
ABZ.EC057	E8 - 5	Ecuador	*Atelopus elegans*	Captive Host	*Blastomonas natatoria*	SUB2577474	KY938126
ABZ.EC060	E8 - 8	Ecuador	*Atelopus elegans*	Captive Host	*Pseudomonas vranovensis*	SUB2577474	KY938127
ABZ.EC061	E8 - 9	Ecuador	*Atelopus elegans*	Captive Host	*Morganella morganii*	SUB2577474	KY938128
ABZ.EC062	E9 - 1	Ecuador	*Atelopus elegans*	Captive Host	*Pseudomonas mosselii*	SUB2577474	KY938129
ABZ.EC063	E9 - 2	Ecuador	*Atelopus elegans*	Captive Host	*Sphingomonas sp.*	SUB2577474	KY938130
ABZ.EC064	E9 - 3	Ecuador	*Atelopus elegans*	Captive Host	*Citrobacter sp.*	SUB2577474	KY938131
ABZ.EC066	E9 - 5	Ecuador	*Atelopus elegans*	Captive Host	*Citrobacter sp.*	SUB2577474	KY938132
ABZ.EC067	E9 - 6	Ecuador	*Atelopus elegans*	Captive Host	*Brevundimonas aurantiaca*	SUB2577474	KY938133
ABZ.EC068	E9 - 7	Ecuador	*Atelopus elegans*	Captive Host	*Blastomonas natatoria*	SUB2577474	KY938134
ABZ.EC069	E9 - 8	Ecuador	*Atelopus elegans*	Captive Host	*Citrobacter sp.*	SUB2577474	KY938135
ABZ.EC070	E9 - 9	Ecuador	*Atelopus elegans*	Captive Host	*Comamonas sp.*	SUB2577474	KY938136
ABZ.EC071	E10 - 1	Ecuador	*Atelopus elegans*	Captive Host	*Comamonas sp.*	SUB2577474	KY938137
ABZ.EC072	E10 - 2	Ecuador	*Atelopus elegans*	Captive Host	*Sphingopyxis sp.*	SUB2577474	KY938138
ABZ.EC073	E10 - 3	Ecuador	*Atelopus elegans*	Captive Host	*Microbacterium foliorum*	SUB2577474	KY938139
ABZ.EC074	E10 - 4	Ecuador	*Atelopus elegans*	Captive Host	*Enterobacter sp.*	SUB2577474	KY938140
ABZ.EC075	E10 - 5	Ecuador	*Atelopus elegans*	Captive Host	*Sphingopyxis sp.*	SUB2577474	KY938141
ABZ.EC076	E11 - 1	Ecuador	*Atelopus elegans*	Captive Host	*Pseudomonas sp.*	SUB2577474	KY938142
ABZ.EC077	E11 - 2	Ecuador	*Atelopus elegans*	Captive Host	*Pseudomonas sp.*	SUB2577474	KY938143
ABZ.EC078	E11 - 3	Ecuador	*Atelopus elegans*	Captive Host	*Pantoea agglomerans*	SUB2577474	KY938144
ABZ.EC079	E11 - 4	Ecuador	*Atelopus elegans*	Captive Host	*Pseudomonas sp.*	SUB2577474	KY938145
ABZ.EC080	E12 - 1	Ecuador	*Atelopus elegans*	Captive Host	*Novosphingobium subterraneum*	SUB2577474	KY938146
ABZ.EC081	E12 - 2	Ecuador	*Atelopus elegans*	Captive Host	*Aeromonas hydrophila*	SUB2577474	KY938147
ABZ.EC082	E12 - 3	Ecuador	*Atelopus elegans*	Captive Host	*Sphingopyxis sp.*	SUB2577474	KY938148
ABZ.EC083	E12 - 4	Ecuador	*Atelopus elegans*	Captive Host	*Tsukamurella sp.*	SUB2577474	KY938149
ABZ.EC085	E12 - 6	Ecuador	*Atelopus elegans*	Captive Host	*Acinetobacter sp.*	SUB2577474	KY938150
ABZ.EC086	E12 - 7	Ecuador	*Atelopus elegans*	Captive Host	*Sphingomonas sp.*	SUB2577474	KY938151
ABZ.EC087	E12 - 8	Ecuador	*Atelopus elegans*	Captive Host	*Sphingopyxis sp.*	SUB2577474	KY938152
ABZ.EC088	E13 - 1	Ecuador	*Atelopus elegans*	Captive Host	*Blastomonas natatoria*	SUB2577474	KY938153
ABZ.EC089	E13 - 2	Ecuador	*Atelopus elegans*	Captive Host	*Pelomonas puraquae*	SUB2577474	KY938154
ABZ.EC090	E13 - 3	Ecuador	*Atelopus elegans*	Captive Host	*Comamonas sp.*	SUB2577474	KY938155
ABZ.EC091	E13 - 4	Ecuador	*Atelopus elegans*	Captive Host	*Sphingomonas sp.*	SUB2577474	KY938156
ABZ.EC092	E13 - 5	Ecuador	*Atelopus elegans*	Captive Host	*Pelomonas puraquae*	SUB2577474	KY938157
ABZ.EC093	E14 - 1	Ecuador	*Atelopus elegans*	Captive Host	*Microbacterium sp.*	SUB2577474	KY938158
ABZ.EC094	E14 - 2	Ecuador	*Atelopus elegans*	Captive Host	*Klebsiella oxytoca*	SUB2577474	KY938159
ABZ.EC095	E14 - 3	Ecuador	*Atelopus elegans*	Captive Host	*Pseudomonas sp.*	SUB2577474	KY938160
ABZ.EC097	E14 - 5	Ecuador	*Atelopus elegans*	Captive Host	*Enterobacter sp.*	SUB2577474	KY938161
ABZ.EC098	E14 - 6	Ecuador	*Atelopus elegans*	Captive Host	*Pseudomonas aeruginosa*	SUB2577474	KY938162
ABZ.EC099	E14 - 7	Ecuador	*Atelopus elegans*	Captive Host	*Pseudomonas sp.*	SUB2577474	KY938163

ABZ.EC100	E14 - 8	Ecuador	*Atelopus elegans*	Captive Host	*Tsukamurella tyrosinosolven*	SUB2577474	KY938164
ABZ.EC101	E14 - 9	Ecuador	*Atelopus elegans*	Captive Host	*Novosphingobium aromaticivoran*	SUB2577474	KY938165
ABZ.EC102	E14 - 10	Ecuador	*Atelopus elegans*	Captive Host	*Aeromonas sp.*	SUB2577474	KY938166
ABZ.EC103	E14 - 11	Ecuador	*Atelopus elegans*	Captive Host	*Pseudomonas putida*	SUB2577474	KY938167
ABZ.EC105	E14 - 13	Ecuador	*Atelopus elegans*	Captive Host	*Novosphingobium subterraneum*	SUB2577474	KY938168
ABZ.EC106	E14 - 14	Ecuador	*Atelopus elegans*	Captive Host	*Sphingobium yanoikuyae*	SUB2577474	KY938169
ABZ.EC107	E15 - 1	Ecuador	*Atelopus elegans*	Captive Host	*Acinetobacter sp.*	SUB2577474	KY938170
ABZ.EC108	E15 - 2	Ecuador	*Atelopus elegans*	Captive Host	*Pelomonas puraquae*	SUB2577474	KY938171
ABZ.EC110	E15 - 4	Ecuador	*Atelopus elegans*	Captive Host	*Sphingomonas sp.*	SUB2577474	KY938172
VF150	A1-1	Colombia	*Atelopus spurrelli*	Captive Host	*Comamonas testosteroni*	SUB2570260	KY910042
VF151	A1-2	Colombia	*Atelopus spurrelli*	Captive Host	*Pseudomonas monteilii*	SUB2570260	KY910043
VF152	A1-4	Colombia	*Atelopus spurrelli*	Captive Host	*Stenotrophomonas sp.*	SUB2570260	KY910044
VF153	A1-5	Colombia	*Atelopus spurrelli*	Captive Host	*Stenotrophomonas maltophilia*	SUB2570260	KY910045
VF154	A1-6	Colombia	*Atelopus spurrelli*	Captive Host	*Chryseobacterium meningoseptic*	SUB2570260	KY910046
VF155	A1-7	Colombia	*Atelopus spurrelli*	Captive Host	*Pseudomonas sp.*	SUB2570260	KY910047
VF156	A1-8	Colombia	*Atelopus spurrelli*	Captive Host	*Stenotrophomonas sp.*	SUB2570260	KY910048
VF157	A1-9	Colombia	*Atelopus spurrelli*	Captive Host	*Pseudomonas sp.*	SUB2570260	KY910049
VF158	A2-1	Colombia	*Atelopus spurrelli*	Captive Host	*Acinetobacter sp.*	SUB2570260	KY910050
VF159	A2-2	Colombia	*Atelopus spurrelli*	Captive Host	*Acinetobacter sp.*	SUB2570260	KY910051
VF160	A2-3	Colombia	*Atelopus spurrelli*	Captive Host	*Acinetobacter sp.*	SUB2570260	KY910052
VF161	A2-4	Colombia	*Atelopus spurrelli*	Captive Host	*Acinetobacter sp.*	SUB2570260	KY910053
VF162	A2-5	Colombia	*Atelopus spurrelli*	Captive Host	*Pseudomonas putida*	SUB2570260	KY910054
VF163	A2-6	Colombia	*Atelopus spurrelli*	Captive Host	*Acinetobacter sp.*	SUB2570260	KY910055
VF164	A4-1	Colombia	*Atelopus spurrelli*	Captive Host	*Chryseobacterium meningoseptic*	SUB2570260	KY910056
VF165	A4-2	Colombia	*Atelopus spurrelli*	Captive Host	*Acinetobacter sp.*	SUB2570260	KY910057
VF166	A4-3	Colombia	*Atelopus spurrelli*	Captive Host	*Acinetobacter sp.*	SUB2570260	KY910058
VF167	A4-4	Colombia	*Atelopus spurrelli*	Captive Host	*Comamonas sp.*	SUB2570260	KY910059
VF168	A4-6	Colombia	*Atelopus spurrelli*	Captive Host	*Chryseobacterium meningoseptic*	SUB2570260	KY910060
VF169	C1-1	Colombia	*Atelopus* aff. *limosus*	Captive Host	*Acinetobacter sp.*	SUB2570260	KY910061
VF170	C1-2	Colombia	*Atelopus* aff. *limosus*	Captive Host	*Bacillus cereus*	SUB2570260	KY910062
VF171	C1-3	Colombia	*Atelopus* aff. *limosus*	Captive Host	*Acinetobacter sp.*	SUB2570260	KY910063
VF172	C1-4	Colombia	*Atelopus* aff. *limosus*	Captive Host	*Listeria sp.*	SUB2570260	KY910064
VF173	C2-1	Colombia	*Atelopus* aff. *limosus*	Captive Host	*Chryseobacterium sp.*	SUB2570260	KY910065
VF174	C2-2	Colombia	*Atelopus* aff. *limosus*	Captive Host	*Acinetobacter sp.*	SUB2570260	KY910066
VF175	C2-3	Colombia	*Atelopus* aff. *limosus*	Captive Host	*Acinetobacter sp.*	SUB2570260	KY910067
VF176	C2-4	Colombia	*Atelopus* aff. *limosus*	Captive Host	*Comamonas testosteroni*	SUB2570260	KY910068
VF177	C2-5	Colombia	*Atelopus* aff. *limosus*	Captive Host	*Pseudomonas putida*	SUB2570260	KY910069
VF178	C2-6	Colombia	*Atelopus* aff. *limosus*	Captive Host	*Stenotrophomonas maltophilia*	SUB2570260	KY910070
VF179	C2-7	Colombia	*Atelopus* aff. *limosus*	Captive Host	*Pseudomonas putida*	SUB2570260	KY910071
VF180	C2-8	Colombia	*Atelopus* aff. *limosus*	Captive Host	*Stenotrophomonas maltophilia*	SUB2570260	KY910072
VF181	C2-9	Colombia	*Atelopus* aff. *limosus*	Captive Host	*Stenotrophomonas maltophilia*	SUB2570260	KY910073
VF182	C2-10	Colombia	*Atelopus* aff. *limosus*	Captive Host	*Chryseobacterium sp.*	SUB2570260	KY910074
VF183	C2-11	Colombia	*Atelopus* aff. *limosus*	Captive Host	*Chryseobacterium indologenes*	SUB2570260	KY910075
VF184	C2-12	Colombia	*Atelopus* aff. *limosus*	Captive Host	*Acinetobacter baumannii*	SUB2570260	KY910076

VF185	C2-13	Colombia	*Atelopus* aff. *limosus*	Captive Host	*Acinetobacter sp.*	SUB2570260	KY910077
VF186	C2-14	Colombia	*Atelopus* aff. *limosus*	Captive Host	*Stenotrophomonas maltophilia*	SUB2570260	KY910078
VF187	C3-1	Colombia	*Atelopus* aff. *limosus*	Captive Host	*Chryseobacterium indologenes*	SUB2570260	KY910079
VF188	C3-2	Colombia	*Atelopus* aff. *limosus*	Captive Host	*Acinetobacter sp.*	SUB2570260	KY910080
VF189	C3-3	Colombia	*Atelopus* aff. *limosus*	Captive Host	*Stenotrophomonas maltophilia*	SUB2570260	KY910081
VF190	C3-4	Colombia	*Atelopus* aff. *limosus*	Captive Host	*Stenotrophomonas maltophilia*	SUB2570260	KY910082
VF191	C3-5	Colombia	*Atelopus* aff. *limosus*	Captive Host	*Acinetobacter calcoaceticus*	SUB2570260	KY910083
VF192	C3-6	Colombia	*Atelopus* aff. *limosus*	Captive Host	*Chryseobacterium sp.*	SUB2570260	KY910084
VF193	C3-7	Colombia	*Atelopus* aff. *limosus*	Captive Host	*Stenotrophomonas maltophilia*	SUB2570260	KY910085
VF194	C3-8	Colombia	*Atelopus* aff. *limosus*	Captive Host	*Stenotrophomonas maltophilia*	SUB2570260	KY910086
VF195	C3-9	Colombia	*Atelopus* aff. *limosus*	Captive Host	*Stenotrophomonas maltophilia*	SUB2570260	KY910087
VF196	C3-11	Colombia	*Atelopus* aff. *limosus*	Captive Host	*Acinetobacter sp.*	SUB2570260	KY910088
VF197	C3-12	Colombia	*Atelopus* aff. *limosus*	Captive Host	*Stenotrophomonas maltophilia*	SUB2570260	KY910089
VF198	C4-2	Colombia	*Atelopus* aff. *limosus*	Captive Host	*Pseudomonas geniculata*	SUB2570260	KY910090
VF199	C4-3	Colombia	*Atelopus* aff. *limosus*	Captive Host	*Comamonas testosteroni*	SUB2570260	KY910091
VF200	C4-4	Colombia	*Atelopus* aff. *limosus*	Captive Host	*Stenotrophomonas sp.*	SUB2570260	KY910092
VF201	C4-5	Colombia	*Atelopus* aff. *limosus*	Captive Host	*Alcaligenes faecalis*	SUB2570260	KY910093
VF202	C4-6	Colombia	*Atelopus* aff. *limosus*	Captive Host	*Dermacoccus sp.*	SUB2570260	KY910094
VF203	C4-7	Colombia	*Atelopus* aff. *limosus*	Captive Host	*Acinetobacter berezinae*	SUB2570260	KY910095
VF204	C4-8	Colombia	*Atelopus* aff. *limosus*	Captive Host	*Stenotrophomonas maltophilia*	SUB2570260	KY910096
VF205	C4-10	Colombia	*Atelopus* aff. *limosus*	Captive Host	*Alcaligenes faecalis*	SUB2570260	KY910097
VF206	C4-11	Colombia	*Atelopus* aff. *limosus*	Captive Host	*Pseudomonas sp.*	SUB2570260	KY910098
VF207	C4-12	Colombia	*Atelopus* aff. *limosus*	Captive Host	*Brevibacterium aureum*	SUB2570260	KY910099
VF208	C5-1	Colombia	*Atelopus* aff. *limosus*	Captive Host	*Kitasatospora phosalacinea*	SUB2570260	KY910100
VF209	C5-2	Colombia	*Atelopus* aff. *limosus*	Captive Host	*Bacillus cereus*	SUB2570260	KY910101
VF210	C5-3	Colombia	*Atelopus* aff. *limosus*	Captive Host	*Stenotrophomonas maltophilia*	SUB2570260	KY910102
VF211	C5-4	Colombia	*Atelopus* aff. *limosus*	Captive Host	*Leucobacter sp.*	SUB2570260	KY910103
VF212	C6-1	Colombia	*Atelopus* aff. *limosus*	Captive Host	*Pseudomonas plecoglossicida*	SUB2570260	KY910104
VF213	C6-2	Colombia	*Atelopus* aff. *limosus*	Captive Host	*Pseudomonas mosselii*	SUB2570260	KY910105
VF214	C6-3	Colombia	*Atelopus* aff. *limosus*	Captive Host	*Pseudomonas putida*	SUB2570260	KY910106
VF215	C6-4	Colombia	*Atelopus* aff. *limosus*	Captive Host	*Pseudomonas sp.*	SUB2570260	KY910107
VF216	C6-5	Colombia	*Atelopus* aff. *limosus*	Captive Host	*Chryseobacterium meningoseptic*	SUB2570260	KY910108
VF217	C6-6	Colombia	*Atelopus* aff. *limosus*	Captive Host	*Pseudomonas putida*	SUB2570260	KY910109
VF218	C6-7	Colombia	*Atelopus* aff. *limosus*	Captive Host	*Pseudomonas putida*	SUB2570260	KY910110
VF219	C7-3	Colombia	*Atelopus* aff. *limosus*	Captive Host	*Bacillus sp.*	SUB2570260	KY910111
VF220	C7-5	Colombia	*Atelopus* aff. *limosus*	Captive Host	*Bacillus sp.*	SUB2570260	KY910112
VF221	C7-6	Colombia	*Atelopus* aff. *limosus*	Captive Host	*Bacillus sp.*	SUB2570260	KY910113
VF222	C7-7	Colombia	*Atelopus* aff. *limosus*	Captive Host	*Bacillus sp.*	SUB2570260	KY910114
VF223	C7-8	Colombia	*Atelopus* aff. *limosus*	Captive Host	*Stenotrophomonas sp.*	SUB2570260	KY910115
