# Supplementary figures and images for: The effect of captivity on the skin microbial symbionts in three Atelopus species from the lowlands of Colombia and Ecuador
Source: PeerJ. 2017 Jul 31;5:e3594. doi: 10.7717/peerj.3594 (PMC5541920; doi:10.7717/peerj.3594)

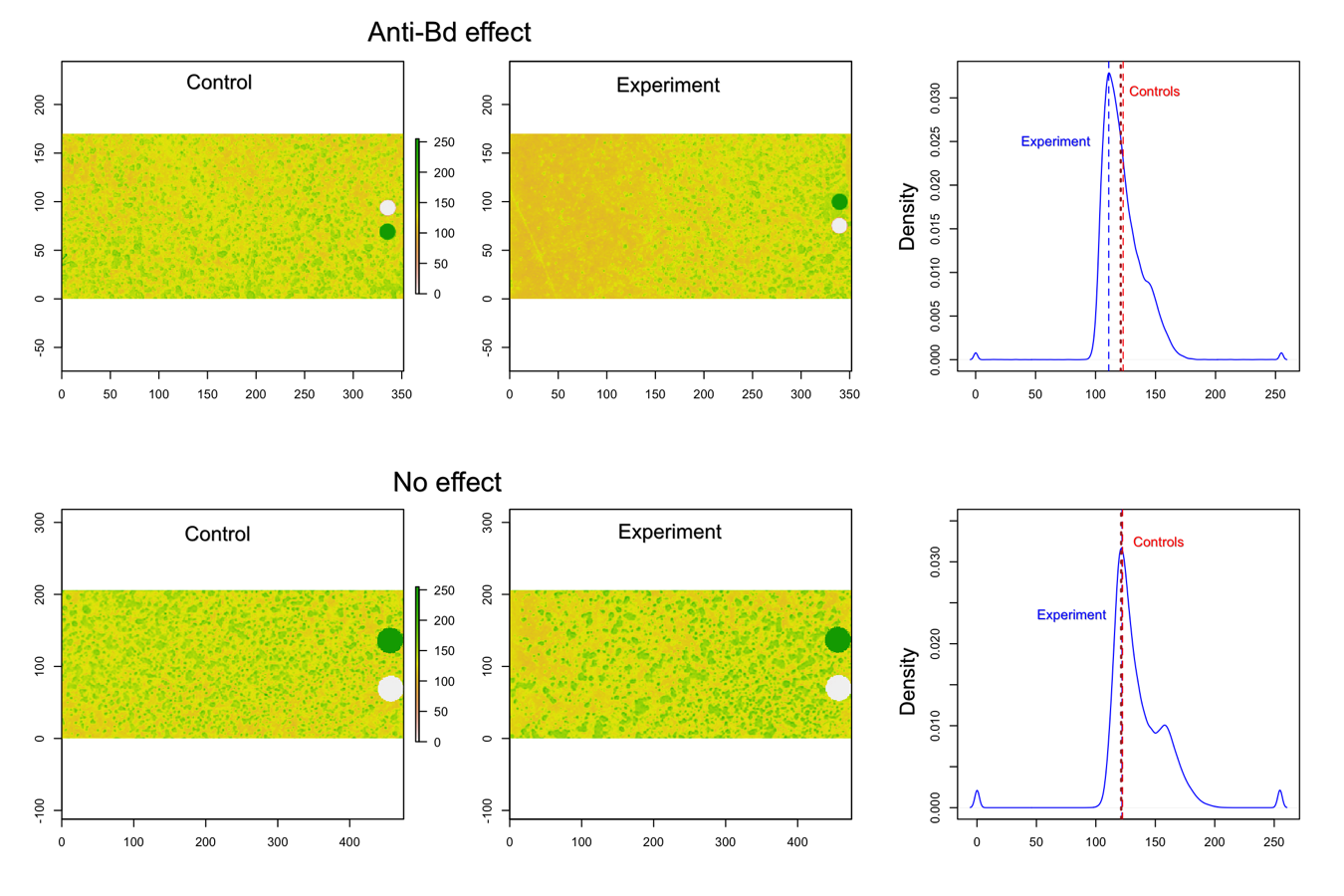

Supplement: Fig. S1 — Control and experimental digital images from two tests: upper panels show an experiment where we visually detected antifungal activity; lower panels show an experiment where tested bacterium did not inhibit Bd growth. On the right, kernel density estimates for color composition in experimental images (solid blue line) and its mode (dashed blue line). Red and brown dashed lines represent the modes of the kernel density estimates for the two assessment methods. [file peerj-05-3594-s002.png]
